# Supplementary material for: Graph theory analysis reveals an assortative pain network vulnerable to attacks
Source: Sci Rep. 2023 Dec 11;13:21985. doi: 10.1038/s41598-023-49458-7 (PMC10713541; doi:10.1038/s41598-023-49458-7)
Supplement: Supplementary file 1 — Supplementary Information. [file 41598_2023_49458_MOESM1_ESM.pdf]

Supplemental Information

Graph theory analysis reveals an assortative pain network vulnerable to attacks

Chong Chen, Adrien Tassou, Valentina Morales and Grégory Scherrer

Table S1. Brain areas in the pain network.

Symbol	Brain area	Group	Group1	Reference
ACAd	Anterior cingulate area, dorsal part	CH	Forebrain	[1–3]
ACAv	Anterior cingulate area, ventral part	CH	Forebrain	[4–6]
SSp	Primary somatosensory area	CH	Forebrain	[1,2]
SSs	Supplementary somatosensory area	CH	Forebrain	[1,2]
MOp	Primary motor area	CH	Forebrain	[1–3]
MOs	Secondary motor area	CH	Forebrain	[1–3]
AI	Agranular insular area	CH	Forebrain	[7–9]
RSPd	Retrosplenial area, dorsal part	CH	Forebrain	[10,11]
RSPv	Retrosplenial area, ventral part	CH	Forebrain	[10,11]
CA1	Field CA1	CH	Forebrain	[12,13]
CA3	Field CA3	CH	Forebrain	[12,13]
DG	Dentate gyrus	CH	Forebrain	[12,13]
CLA	Claustrum	CH	Forebrain	[12–14]
BLA	Basolateral amygdala nucleus	CH	Forebrain	[15–18]
CP	Caudoputamen	CH	Forebrain	[19,20]
ACB	Nucleus accumbens	CH	Forebrain	[21–23]
AAA	Anterior amygdala area	CH	Forebrain	[15,17,18]
CEA	Central amygdala nucleus	CH	Forebrain	[15,18,24,25]
VAL	Ventral anterior-lateral complex of the thalamus	BS	Forebrain	[26,27]
VM	Ventral medial nucleus of the thalamus	BS	Forebrain	[26,27]
VPL	Ventral posterolateral nucleus of the thalamus	BS	Forebrain	[26,27]
VPM	Ventral posteromedial nucleus of the thalamus	BS	Forebrain	[26,27]
LP	Lateral posterior nucleus of the thalamus	BS	Forebrain	[26,27]
PO	Posterior complex of the thalamus	BS	Forebrain	[26,27]
AV	Anteroventral nucleus of thalamus	BS	Forebrain	[26,27]

AM	Anteromedial nucleus of thalamus	BS	Forebrain	[26,27]
LD	Lateral dorsal nucleus of thalamus	BS	Forebrain	[26,27]
MD	Mediodorsal nucleus of thalamus	BS	Forebrain	[26,27]
PVT	Paraventricular nucleus of the thalamus	BS	Forebrain	[28–30]
PT	Parataenial nucleus	BS	Forebrain	[26,27]
MH	Medial habenula	BS	Forebrain	[28]
LH	Lateral habenula	BS	Forebrain	[28,29]
ZI	Zona incerta	BS	Midbrain	[31–33]
PB	Parabrachial nucleus	BS	Midbrain	[34–36]
PG	Pontine gray	BS	Midbrain	
RN	Red nucleus	BS	Midbrain	[37,38]
RPO	Nucleus raphe pontis	BS	Midbrain	[37,38]
PAG	Periaqueductal gray	BS	Midbrain	[39]
CENT	Central lobule	CB	Hindbrain	[40,41]
CUL	Culmen	CB	Hindbrain	[40,41]
PYR	Pyramus (VIII)	CB	Hindbrain	[40,41]
NOD	Nodulus (X)	CB	Hindbrain	[40,41]
SIM	Simple lobule	CB	Hindbrain	[40,41]
AN	Ansiform lobule	CB	Hindbrain	[40,41]
PRM	Paramedian lobule	CB	Hindbrain	[40,41]
PFL	Paraflocculus	CB	Hindbrain	[40,41]
FN	Fastigial nucleus	CB	Hindbrain	[40,41]
IP	Interposed nucleus	CB	Hindbrain	[40,41]

Table S2. Centrality of all brain areas in the pain network.

Vertex	Degree	Closeness	Betweenness
ACAd	45	0.17609827	1
ACAv	41	0.16582495	119
SSp	51	0.22318075	0
SSs	38	0.20510836	86
MOp	49	0.22807327	166
MOs	55	0.21569048	154
AI	39	0.16804397	93
RSPd	28	0.1711025	3
RSPv	37	0.15562876	40
CA1	36	0.15403277	0
CA3	21	0.18777668	82
DG	15	0.17941749	0
CLA	25	0.19679365	0
BLA	31	0.17118668	29
CP	58	0.21879034	216
ACB	34	0.19954181	129
AAA	17	0.19487944	24
CEA	36	0.22793885	318
VAL	30	0.17249759	12
VM	37	0.21124779	53
VPL	28	0.18454415	21
VPM	21	0.19529003	2
LP	37	0.18470254	32
PO	31	0.19807701	53
AV	22	0.12558275	0
AM	30	0.21195561	100

LD	25	0.16324199	45
MD	51	0.22575112	215
PVT	24	0.20913698	58
PT	18	0.15850886	57
MH	9	0.11750354	0
LH	15	0.18063207	0
ZI	50	0.20198961	315
PB	25	0.229578	730
PG	16	0.15370687	43
RN	24	0.171889	38
RPO	18	0.08327624	0
PAG	37	0.17434472	103
CENT	3	0.04748381	0
CUL	5	0.09123744	0
PYR	1	0.10607869	0
NOD	3	0.06280077	0
SIM	6	0.09244126	134
AN	7	0.09143168	112
PRM	4	0.11274039	0
PFL	8	0.09773672	1
FN	7	0.15933981	235
IP	8	0.08289321	294
IO	8	0.20393627	47

Table S3. Community of each brain area in the pain network.

Vertex	Community No.
SIM	1
IP	1
AN	2
PRM	2
PFL	2
IO	2
ACAd	3
ACAv	3
MOs	3
AI	3
RSPd	3
RSPv	3
CLA	3
BLA	3
CP	3
AAA	3
CEA	3
VM	3
LP	3
AV	3
AM	3
LD	3
MD	3
ZI	3

RN	3
PAG	3
ACB	4
PVT	4
PT	4
RPO	4
CA1	5
CA3	5
DG	5
MH	5
LH	5
SSp	6
SSs	6
MOp	6
VAL	6
VPL	6
VPM	6
PO	6
PB	7
PG	8
CENT	9
CUL	10
PYR	11
NOD	12
FN	13

Reference:

1. Vierck, C.J., Whitsel, B.L., Favorov, O.V., Brown, A.W., and Tommerdahl, M. (2013). Role of primary somatosensory cortex in the coding of pain. Pain *154*, 334–344.

2. Bushnell, M.C., Duncan, G.H., Hofbauer, R.K., Ha, B., Chen, J.I., and Carrier, B. (1999). Pain perception: is there a role for primary somatosensory cortex? Proc. Natl. Acad. Sci. U. S. A. *96*, 7705–7709.

3. Leite, J., Carvalho, S., Battistella, L.R., Caumo, W., and Fregni, F. (2017). Editorial: The Role of Primary Motor Cortex as a Marker and Modulator of Pain Control and Emotional-Affective Processing. Front. Hum. Neurosci. *11*, 270.

4. Hutchison, W.D., Davis, K.D., Lozano, A.M., Tasker, R.R., and Dostrovsky, J.O. (1999). Pain-related neurons in the human cingulate cortex. Nat. Neurosci. *2*, 403–405.

5. Fuchs, P.N., Peng, Y.B., Boyette-Davis, J.A., and Uhelski, M.L. (2014). The anterior cingulate cortex and pain processing. Front. Integr. Neurosci. *8*, 35.

6. Bliss, T.V.P., Collingridge, G.L., Kaang, B.-K., and Zhuo, M. (2016). Synaptic plasticity in the anterior cingulate cortex in acute and chronic pain. Nat. Rev. Neurosci. *17*, 485–496.

7. Starr, C.J., Sawaki, L., Wittenberg, G.F., Burdette, J.H., Oshiro, Y., Quevedo, A.S., and Coghill, R.C. (2009). Roles of the insular cortex in the modulation of pain: insights from brain lesions. J. Neurosci. *29*, 2684–2694.

8. Lu, C., Yang, T., Zhao, H., Zhang, M., Meng, F., Fu, H., Xie, Y., and Xu, H. (2016). Insular Cortex is Critical for the Perception, Modulation, and Chronification of Pain. Neurosci. Bull. *32*, 191–201.

9. Mutschler, I., Ball, T., Wankerl, J., and Strigo, I.A. (2012). Pain and emotion in the insular cortex: evidence for functional reorganization in major depression. Neurosci. Lett. *520*, 204–209.

10. Barrière, D.A., Hamieh, A.M., Magalhães, R., Traoré, A., Barbier, J., Bonny, J.-M., Ardid, D., Busserolles, J., Mériaux, S., and Marchand, F. (2019). Structural and functional alterations in the retrosplenial cortex following neuropathic pain. Pain *160*, 2241–2254.

11. Wik, G., Fischer, H., Finer, B., Bragee, B., Kristianson, M., and Fredrikson, M. (2006). Retrospenial cortical deactivation during painful stimulation of fibromyalgic patients. Int. J. Neurosci. *116*, 1–8.

12. Mutso, A.A., Radzicki, D., Baliki, M.N., Huang, L., Banisadr, G., Centeno, M.V., Radulovic, J., Martina, M., Miller, R.J., and Apkarian, A.V. (2012). Abnormalities in hippocampal functioning with persistent pain. J. Neurosci. *32*, 5747–5756.

13. Grilli, M. (2017). Chronic pain and adult hippocampal neurogenesis: translational implications from preclinical studies. J. Pain Res. *10*, 2281–2286.

14. Gracely, R.H., Geisser, M.E., Giesecke, T., Grant, M.A.B., Petzke, F., Williams, D.A., and Clauw, D.J. (2004). Pain catastrophizing and neural responses to pain among persons with fibromyalgia. Brain *127*, 835–843.

15. Neugebauer, V. (2015). Amygdala pain mechanisms. Handb. Exp. Pharmacol. *227*, 261–284.

16. Corder, G., Ahanonu, B., Grewe, B.F., Wang, D., Schnitzer, M.J., and Scherrer, G. (2019). An amygdalar neural ensemble that encodes the unpleasantness of pain. Science *363*, 276–281.

17. Li, Z., Wang, J., Chen, L., Zhang, M., and Wan, Y. (2013). Basolateral amygdala lesion inhibits the development of pain chronicity in neuropathic pain rats. PLoS One *8*, e70921.

18. Thompson, J.M., and Neugebauer, V. (2017). Amygdala Plasticity and Pain. Pain Res. Manag. *2017*, 8296501.

19. Barceló, A.C., Filippini, B., and Pazo, J.H. (2012). The striatum and pain modulation. Cell. Mol. Neurobiol. *32*, 1–12.

20. Borsook, D., Upadhyay, J., Chudler, E.H., and Becerra, L. (2010). A key role of the basal ganglia in pain and analgesia--insights gained through human functional imaging. Mol. Pain *6*, 27.

21. Baliki, M.N., Geha, P.Y., Fields, H.L., and Apkarian, A.V. (2010). Predicting value of pain and analgesia: nucleus accumbens response to noxious stimuli changes in the presence of chronic pain. Neuron *66*, 149–160.

22. Harris, H.N., and Peng, Y.B. (2020). Evidence and explanation for the involvement of the nucleus accumbens in pain processing. Neural Regeneration Res. *15*, 597–605.

23. DosSantos, M.F., Moura, B. de S., and DaSilva, A.F. (2017). Reward Circuitry Plasticity in Pain Perception and Modulation. Front. Pharmacol. *8*, 790.

24. Warlow, S.M., Naffziger, E.E., and Berridge, K.C. (2020). The central amygdala recruits mesocorticolimbic circuitry for pursuit of reward or pain. Nat. Commun. *11*, 2716.

25. O'Neill, P.-K., and Meszaros, J. (2020). Chronic Pain Releases Parabrachial Activity from Central Amygdala Inhibition. J. Neurosci. *40*, 7996–7998.

26. Gustin, S.M., Peck, C.C., Wilcox, S.L., Nash, P.G., Murray, G.M., and Henderson, L.A. (2011). Different pain, different brain: thalamic anatomy in neuropathic and non-neuropathic chronic pain syndromes. J. Neurosci. *31*, 5956–5964.

27. Sotgiu, M.L. (2001). The Thalamus and Pain. In Neuroscience: Focus on Acute and Chronic Pain (Springer Milan), pp. 37–42.

28. Shelton, L., Becerra, L., and Borsook, D. (2012). Unmasking the mysteries of the habenula in pain and analgesia. Prog. Neurobiol. *96*, 208–219.

29. Shelton, L., Pendse, G., Maleki, N., Moulton, E.A., Lebel, A., Becerra, L., and Borsook, D. (2012). Mapping pain activation and connectivity of the human habenula. J. Neurophysiol. *107*, 2633–2648.

30. Kang, S., Li, J., Zuo, W., Chen, P., Gregor, D., Fu, R., Han, X., Bekker, A., and Ye, J.-H. (2019). Downregulation of M-channels in lateral habenula mediates hyperalgesia during alcohol withdrawal in rats. Sci. Rep. *9*, 2714.

31. Lu, C.W., Harper, D.E., Askari, A., Willsey, M.S., Vu, P.P., Schrepf, A.D., Harte, S.E., and Patil, P.G. (2021). Stimulation of zona incerta selectively modulates pain in humans. Sci. Rep. *11*, 8924.

32. Moon, H.C., and Park, Y.S. (2017). Reduced GABAergic neuronal activity in zona incerta causes neuropathic pain in a rat sciatic nerve chronic constriction injury model. J. Pain Res. *10*, 1125–1134.

33. Masri, R., Quiton, R.L., Lucas, J.M., Murray, P.D., Thompson, S.M., and Keller, A. (2009). Zona incerta: a role in central pain. J. Neurophysiol. *102*, 181–191.

34. Chiang, M.C., Bowen, A., Schier, L.A., Tupone, D., Uddin, O., and Heinricher, M.M. (2019). Parabrachial Complex: A Hub for Pain and Aversion. J. Neurosci. *39*, 8225–8230.

35. Roeder, Z., Chen, Q., Davis, S., Carlson, J.D., Tupone, D., and Heinricher, M.M. (2016). Parabrachial complex links pain transmission to descending pain modulation. Pain *157*, 2697–2708.

36. Palmiter, R.D. (2018). The Parabrachial Nucleus: CGRP Neurons Function as a General Alarm. Trends Neurosci. *41*, 280–293.

37. Yang, Q.-Q., Li, H.-N., Zhang, S.-T., Yu, Y.-L., Wei, W., Zhang, X., Wang, J.-Y., and Zeng, X.-Y. (2020). Red nucleus IL-6 mediates the maintenance of neuropathic pain by inducing the productions of TNF-α and IL-1β through the JAK2/STAT3 and ERK signaling pathways. Neuropathology. 10.1111/neup.12653.

38. François, A., Low, S.A., Sypek, E.I., Christensen, A.J., Sotoudeh, C., Beier, K.T., Ramakrishnan, C., Ritola, K.D., Sharif-Naeini, R., Deisseroth, K., et al. (2017). A Brainstem-Spinal Cord Inhibitory Circuit for Mechanical Pain Modulation by GABA and Enkephalins. Neuron *93*, 822–839.e6.

39. Mokhtar, M., and Singh, P. (2020). Neuroanatomy, Periaqueductal Gray. In StatPearls (StatPearls Publishing).

40. Moulton, E.A., Schmahmann, J.D., Becerra, L., and Borsook, D. (2010). The cerebellum and pain: passive integrator or active participator? Brain Res. Rev. *65*, 14–27.

41. Coombes, S.A., and Misra, G. (2016). Pain and motor processing in the human cerebellum. Pain *157*, 117–127.
